# Trends of and Factors Associated with Maternal Near-Miss in Selected Hospitals in North Shewa Zone, Central Ethiopia

**DOI:** 10.1155/2022/2023652

**Published:** 2022-09-09

**Authors:** Tesfay Brhane Gebremariam, Takele Gezahegn Demie, Behailu Tariku Derseh, Kalayu Brhane Mruts

**Affiliations:** ^1^Department of Public Health, College of Health Science, Debre Berhan University, Debre Berhan, Ethiopia; ^2^School of Public Health, Saint Paul's Hospital Millennium Medical College, Addis Ababa, Ethiopia; ^3^Curtin School of Population Health, Curtin University, Western Australia, Perth, Australia

## Abstract

Maternal near-miss (MNM) refers to a woman who nearly died but survived a complication that occurred during pregnancy, childbirth, or within 42 days of termination of pregnancy. Studies in Ethiopia showed an inconsistent proportion of MNM across time and in different setups. This study is aimed at assessing the magnitude, trends, and correlates of MNM at three selected hospitals in North Shewa Zone, Central Ethiopia. A hospital-based cross-sectional study was conducted among 905 mothers who gave birth from 2012 to 2017 in three hospitals using the WHO criteria for MNM. Medical records of the study subjects were selected using a systematic sampling technique. Data were retrieved using a pretested data extraction tool. Association between MNM and independent variables was assessed by using a binary logistic regression model. An odds ratio with a 95% confidence interval (CI) and *p* value of <0.05 were used to declare the level of significance. Of the 905 medical records reviewed, the prevalence of MNM was 14.3% (95%CI = 11.9 − 16.6) and similar over the last six years (2012-2017). The magnitude of life-threatening pregnancy complications was found to be 12.7%; severe preeclampsia (31%) and postpartum hemorrhage (26%) account for the highest proportion. Admission at a higher level of obstetric care like referral hospital (AOR = 4.85; 95% CI: 1.82-12.94) and general hospital (AOR = 3.76; 95% CI: 1.37-10.33), not using partograph for labor monitoring (AOR = 1.89; 95% CI: 1.17-3.04), history of abortion (AOR = 2.52; 95% CI: 1.18-5.37), and any other pregnancy complications (AOR = 6.91; 95% CI: 3.89-12.28) were factors significantly associated with higher MNM. Even though lower than the national figure, the proportion of MNM in the study area was very high, and there were no significant changes over the last six consecutive years. Giving special emphasis to women with prior history of pregnancy complications, hypertensive disorders of pregnancy, and obstetric hemorrhage with strict and quick management protocols and the use of partograph for labor monitoring are recommended to reduce the burden of severe maternal outcomes in the study area and Ethiopia.

## 1. Introduction

Although most pregnancies and childbirths are ordinary with good maternal and perinatal outcomes, approximately 15% of all pregnant women develop potentially life-threatening complications including death [1]. A maternal near-miss (MNM) case is defined as “a woman who nearly died but survived a complication(s) that occurred during pregnancy, childbirth, or within 42 days of termination of pregnancy” [2, 3]. Most of these complications can be preventable or treatable [2]. Maternal mortality and morbidity remain unacceptably high in developing countries, where 99% of maternal death occurs. The risk of maternal death is 1 in 41 and 1 in 3300 live births in developing and developed countries, respectively [1–3].

The presence of life-threatening complications during pregnancy, indications of organ dysfunction, or clinical interventions for managing these complications including blood transfusion, interventional radiology, laparotomy/hysterectomy, and other emergency surgical interventions, excluding cesarean section, could be used to detect maternal near-miss [2, 3].

Nowadays, MNM was considered a vital indicator used to monitor maternal health in general and the quality of obstetric services in particular. It is a useful indicator set by the World Health Organization (WHO) to identify the leading pregnancy complications, underlining risk factors, interventions that did and did not work, and the availability of life-saving obstetric services in an obstetric care unit [2]. These life-threatening complications not only increase the risk of maternal death but also increase the probability of a bad perinatal outcome and the presence of MNM conditions in women as an independent risk factor for adverse perinatal outcomes [4].

A systematic review by WHO reported that individual prevalence rates for severe acute maternal morbidity (SAMM) or near-miss range from 0.8% to 8.23% in disease-specific criteria while ranging from 0.38% to 1.09% for organ system-based criteria and 0.01% to 0.99% in studies using management-based criteria [5]. A systematic review by Sikder et al. showed that the weighted pooled worldwide prevalence of MNM was 18.67/1000 with a large volume of heterogeneity. A study in Bangladesh reported that 25% of women had at least one pregnancy complication, while 2% of women experienced near-misses [6].

Among the studies that had been conducted in Africa, the weighted pooled prevalence of MNM was 31.88% with significant heterogeneity between studies [7]. Sub-Saharan Africa (SSA) alone shoulders more than 66% of global maternal deaths [1]. In addition, very high incidence and prevalence ratio and case fatality ratio of MNM were reported by studies from sub-Saharan Africa, which ranges from 1.1% to 10.1% [8].

One of the nations with the highest rates of maternal morbidity and mortality is Ethiopia. The Ethiopian Public Health Institute's research of a nationwide dataset revealed that there were 67,567 MNM cases, with a crude MNM incidence ratio of 20.8% (9.1-38.8%) and a mortality index of 0.64% (435/68,002) for the direct causes of maternal mortality. Incidence of MNM, mortality index, and the provision of signal functions of emergency obstetric care all showed considerable geographical variation [9]. According to the Ethiopian Demographic and Health Survey (EDHS) reports, the pregnancy-related mortality ratio decreased from 871 in 2000 to 412 in 2016, but with slow progress and large confidence intervals [10]. Different studies in Ethiopia also showed different findings. A study in Debre Markos Hospital revealed 29.7% of near-miss cases with a decreased trend of MNM ratio from 50% to 34% during the last five years before the study [11], while another study also reported 26.6% prevalence of MNM [12].

While ruptured uterus, sepsis, obstructed labor, and hemorrhage were reported as the most common morbidities [8], obstructed labor and hemorrhage were the most common MNM events, and septic abortion and sepsis were the least prevalent causes [11]. A study in Gurage Zone, Southern Ethiopia, revealed that prior history of cesarean section, being referred from other health facilities, and first delay were factors associated with MNM [13]. Pregnancy complications were significantly higher in women with biological risk factors like age less than 18 and greater than 35, a history of stillbirth or miscarriage, and nulliparity [6]. Maternal morbidity is still a tragedy in Ethiopia despite the increased global attention and national efforts to reduce it.

The WHO and partners published a consensus statement and full strategy paper on ending preventable maternal death (EPMM) in anticipation of the launch of the SDGs. The EPMM target for reducing the global maternal mortality ratio (MMR) by 2030 was adopted as SDG target 3.1 to reduce global MMR to less than 70 per 100 000 live births by 2030; Ethiopia also intends to achieve this target by 2030 [1].

A small number of research [4, 9, 11–13] on the prevalence of severe pregnancy complications (near-miss cases), their trend, and determining factors were completed in Ethiopia at the time this study was initiated. These studies have reported inconsistent findings. Despite this, there is no similar study conducted in the study area. Thus, it was deemed vital to conduct this research to verify this finding and provide current evidence in a more varied way. Therefore, the purpose of this study was to assess the prevalence of maternal near-miss, as well as its trends and contributing factors, among women who have been admitted to maternity services in selected hospitals in the North Shewa Zone of Central Ethiopia. Health facilities, clinicians, and service units might use the findings of this study to identify and prioritize the leading life-threatening pregnancy complications, which could, in turn, inform high-level decisions and programs which improve life-saving obstetric service delivery systems.

## 2. Materials and Methods

### 2.1. Study Design, Areas, and Period

A hospital-based cross-sectional study was conducted among mothers who gave birth from 2012 to 2017 in three selected hospitals in North Shewa Zone, Central Ethiopia. The data for this study was collected in January 2018. North Shewa Zone, located in the South-Eastern direction of Amhara Regional State, has more than 2.3 million populations. Debre Berhan town, located 130 kilometers away from Addis Ababa, is the capital city of the zonal administration. The zone has a total of 10 and 97 functional hospitals and health centers, respectively. There were three selected hospitals, namely, Debre Berhan Referral Hospital, Enat General Hospital, and Mehal Meda Primary (District) Hospital. Debre Berhan Referral Hospital, located in Debre Berhan town, was the central referral center of the zonal health system. It provides preventive, curative, and rehabilitative services including comprehensive pregnancy and newborn care. Enat General Hospital was located 133 kilometers east of Debre Berhan town and served as a general hospital for around five districts with a special emphasis on obstetric services. Mehal Meda Primary Hospital was located 150 kilometers north of Debre Berhan town and served as a primary care provision center with basic and emergency obstetric care including the emergency caesarian section [14].

### 2.2. Study Subject, Sample Size, and Sampling Procedure

Mothers who were admitted for delivery or other obstetric services in those selected hospitals were taken as the study population. Three (3) out of the ten (10) hospitals were selected purposively based on the level of obstetric care they provide (as they represent referral, general, and primary hospitals) and the number of women who gave birth. The sample size for this study was 941 women, which was calculated based on the following assumptions: 95% confidence level, 3% margin of error, and 30% proportion of severe pregnancy complications that have been taken from the study conducted in Debre Markos Referral Hospital [11] and 5% incomplete rate of records.

This total sample size was allocated to three hospitals proportional to the average annual number of delivery. The sample size per hospital was again distributed over six years proportional to the annual number of births per hospital. In each hospital, the participant women were selected by using a systematic sampling technique using the women's medical card registration number from the delivery registration book as a sampling frame. If the selected medical registration number is missed in the card room, a replacement was done with the next serial registration number. Medical records that fulfill minimum inclusion criteria were included. Medical charts of mothers that contain at least a diagnosis at admission, mode of delivery, treatments, or care given to the women during that pregnancy or childbirth were included in the study.

### 2.3. Study Variables

The primary outcome of this research is the prevalence of MNM, computed as the total number of MNM cases per total number of deliveries from January 2012 to December 2017. The secondary outcomes include the trends in the prevalence of MNM across the six years. On the other hand, maternal age, parity, gravidity, current, and previous reproductive history were taken as independent variables.

### 2.4. Operational Definitions

In this study, maternal death (MD) is defined as death while pregnant or within 42 days of the end of pregnancy, from any cause related to or aggravated by the pregnancy or its management, but not from accidental or incidental causes.

Maternal near-miss (MNM) refers to a woman who nearly died but survived a complication that occurred during pregnancy, childbirth, or within 42 days of termination of pregnancy.

Prevalence of maternal near-miss refers to the number of MNM cases per 100 deliveries during a specified period.

### 2.5. Data Collection and Analyses Methods

The data for this study were collected from the medical record of women who gave birth in three selected hospitals from January 2012 to December 2017 using a pretested structured data extraction tool and the WHO near-miss identification [2, 3]. The tool contains maternal age, previous reproductive history including history of pregnancy complications, and current obstetric history like antenatal care (ANC) visits, place of delivery, a birth attendant at delivery, mode of delivery, referral history, and partograph use during delivery. The MNM was diagnosed using the WHO near-miss criteria [3]. This criterion has a set of life-threatening pregnancy complications, organ dysfunction(s), underlining conditions, and life-saving clinical interventions. The data extraction tool was filled by trained midwives and supervised by the investigators.

Data were cleaned and checked manually before being entered into the software and then entered into Epi Info version 7. After checking for completeness and cleanness using frequencies, the data was analyzed using SPSS version 20. Descriptive statistics such as frequencies and proportions were performed, and missing values were managed. Association between MNM and the independent variables was assessed by using the binary logistic regression model. Variables having a *p* value < 0.05 at the bivariate logistic regression and the biological variable age were entered into multivariable binary logistic regressions for controlling confounding variables. Collinearity among variables was checked using the variance inflation factor. An odds ratio with a 95% confidence interval was used to present the data. The significance level was considered at a *p* value of <0.05. Finally, the results are presented using tables and figures along with text descriptions.

## 3. Results

### 3.1. Sociodemographic and Reproductive History of Study Mothers

From the total 941 medical records or patient cards reviewed, 905 medical records fully filed the inclusion criteria by completeness and were included in the analysis. About half (49.9%) of medical records were from Debre Berhan Referral Hospital, while 288 (31.8%) and 165 (18.2) were from Enat and Mehal Meda hospitals, respectively. The majority (73.7%) of the mothers were in the age range of 20-34 during the time of childbirth. Nearly half of the mothers (48.3%) were nulliparous with the median parity of one child. About 4.3%, 6%, and 8.5% of the mothers had a previous history of stillbirth, abortion, or any other pregnancy complications. More than two-thirds of the mothers (68.4%) had antenatal care (ANC) visits, and 97.3% of their labor was attended by skilled birth attendants. Partograph was used to monitor labor only for 202 (22.8%) study subjects.  Table 1 describes the reproductive history of the study subjects.

### 3.2. Prevalence of Life-Threatening Pregnancy Complications and Maternal Near-Miss

In this study, more than half (56%) of the mothers were admitted with normal labor without any complications; however, 44% of the participants had developed complications during their delivery. Of those who developed complications, 31.7% had maternal and 6.9% perinatal complications, while the rest 5.4% developed both maternal and perinatal complications.

The magnitude of the maternal near-miss (MNM) was computed from the life-threatening pregnancy complications, organ dysfunction, and life-saving criteria. Hence, of the 905 participants, 129 (14.3%) (95%CI = 11.9 − 16.6) had developed maternal near-miss conditions during their delivery. The magnitude of life-threatening pregnancy complications was found to be 12.7%, which contributed to a significant proportion of severe maternal morbidity. Moreover, the magnitude of MNM using organ dysfunction and life-saving criteria was relatively low (i.e., 4% for each).

Severe preeclampsia (4.6%) and severe postpartum hemorrhage (3.9%) were found to be the leading life-threatening pregnancy complications. Eclampsia, uterine rupture, and sepsis had a prevalence of 2.3%, 2.2%, and 1.8%, respectively. Blood transfusion (2.4%) and laparotomy (1.5%) were the commonest pregnancy interventions, while interventional radiology (0.4%) was rarely performed. Cardiovascular (1.8%) and coagulation (1.2%) dysfunctions were the commonly reported complications, while hepatic dysfunction (0.3%) was observed rarely.

**Figure 1:**
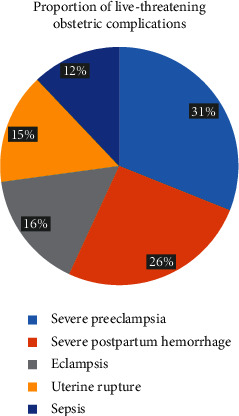
Six-year (2012-2017) average proportion of life-threatening pregnancy complications among mothers admitted in selected hospitals of North Shewa Zone, Central Ethiopia.

Of the total pregnancy complications recorded, severe preeclampsia takes the highest proportion (31.3%) followed by severe postpartum hemorrhage (26.1%). As shown in Figure 1, complications of hypertensive disorder during pregnancy (severe preeclampsia and eclampsia together) accounted for nearly half (47%) of the total life-threatening pregnancy complications (Figure 1).

### 3.3. Trends of Maternal Near-Miss (from 2012 to 2017)

**Figure 2:**
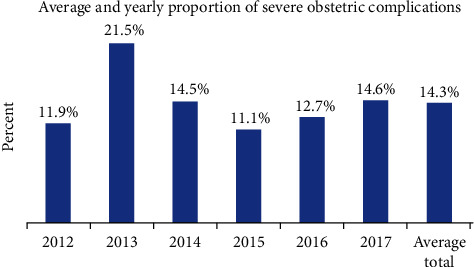
Yearly prevalence of near-miss cases among mothers admitted in selected hospitals of North Shewa Zone, Central Ethiopia, from 2012 to 2017 (*n* = 129).

This study revealed also that, except in 2013 when MNM reached its peak of 21.5%, there is no significant change (chi − square = 8.7, *p* value = 0.12) in the magnitude of the MNM over the last six-year period (2012-2017) (Figure 2). Even though not statistically significant, the prevalence of severe preeclampsia increased from 2.4% in 2012 to more than 5% in 2014 through 2016 and then slightly reduces to 4.2% in 2017.

From the 905 participants randomly selected in the last six consecutive years, seven maternal deaths were reported providing a maternal mortality ratio of 773 per 100, 000 live births. The MNM to mortality ratio was estimated to be 18.4. The maternal mortality index, estimated as the total number of maternal deaths per total number of severe maternal outcomes, was found to be 0.05; i.e., only 5% of women who suffer from severe maternal complications died, while the rest were saved because of life-saving obstetric interventions.

### 3.4. Factors Associated with Maternal Near-Miss

Using bivariate regression analysis, the hospital where mothers were admitted, gravidity, parity, history of any previous pregnancy complications, previous history of cesarean section, stillbirth, history of abortion, and use of partograph during childbirth were significantly associated with MNM. After testing for multicollinearity, a multivariable logistic regression model was used to control potential confounding variables. The result of multivariable logistic regression indicated that the site and type of the hospital, history of abortion, history of any pregnancy complications, and use of partograph have remained the associated factors with MNM at *p* value < 0.05.

Mothers who were admitted to Debre Berhan Referral Hospital were almost five times more likely to develop MNM as compared to participants who were admitted to Mehal Meda Primary Hospital (AOR = 4.85; 95%CI = 1.82, 12.94). Similarly, participants who were admitted to Enat General Hospital were almost four times more likely to develop MNM as compared to participants who were admitted to Mehal Meda Primary Hospital (AOR = 3.76; 95%CI = 1.37, 10.33). Participants whose labor was followed without the use of a partograph were two times more likely to develop MNM than those whose labor was followed using a partograph (AOR = 1.89; 95%CI = 1.17, 3.04). Participants who had a history of abortion were 2.5 times more likely to develop MNM as compared to participants who had no history of abortion (AOR = 2.52; 95%CI = 1.18, 5.37). Furthermore, participants who had a history of pregnancy complications were seven times more likely to develop MNM as compared to participants who had no history of pregnancy complications (AOR = 6.91; 95%CI = 3.89, 12.28) (Table 2).

## 4. Discussion

This study is aimed at assessing the magnitude of MNM and its associated factors at selected hospitals in North Shewa Zone, Central Ethiopia, from 2012 to 2017. In this study, the proportion of MNM was found to be 14.3%. This magnitude of maternal morbidity implies that unless the health facilities provide critical life-saving interventions, maternal mortality will be extremely higher than what is known for Ethiopia so far. The majority of life-threatening pregnancy complications are preventable by providing quality antenatal follow-up and providing essential obstetric care during labor and delivery. However, this high occurrence of near-miss cases suggests that these facilities are not providing sufficient preventive maternity services.

This study reported that there was no significant change in the prevalence of MNM over the last six-year (2012 to 2017) trend. This suggests that despite efforts made so far to prevent pregnancy complications, the occurrence of life-threatening pregnancy complications remains the same over the six years. A potential increase in facility delivery, improvements in the registration of clients' medical history, and a potential increase in the prevalence of hypertension in the general population and among pregnant mothers due to lifestyle changes could justify the lack of reduction in the proportion of pregnancy complications across the six years. Putting in mind the difference in the quality of patient records from time to time, it could be noted that preventive obstetric care has not shown improvement in the last six years, while there may be good progress in the provision of curative obstetric care. Even though there is a difference in the time frame, the study done at Debre Markos Referral Hospital showed a significant reduction in MNM from 2008 to 2012 [11].

Many works of literature so far did not report a trend or average prevalence of near-miss (rather they report a single year or point prevalence) which makes it difficult to compare with this study. Only one study done in Debre Markos Referral Hospital [11] reported an average (2008 to 2012) prevalence of 29.7%, which is two times higher than the finding of this research. This difference is possibly due to the difference in the study period and facility types such that the current study includes a district hospital that potentially has low cases of near-miss. However, this finding is comparable with the finding of studies conducted in the Gondar University Referral Hospital in 2019 [15] which reported a 15.8% proportion of MNM at maternity wards and that of Jimma University Teaching Hospital, South West Ethiopia [16].

However, the prevalence of near-miss reported in our study is lower than that reported by studies in selected hospitals of Amhara regional state (26.6%) [12], South West Ethiopia (24.85%) [17] and a study done in a Tertiary Care Hospital of India [18]. It is also much lower than the findings done in Hawasa and Yrgalem hospitals of Southern Ethiopia [19]. On the contrary, the prevalence of MNM in this study was higher than in studies done in Hiwot Fana and Jugal hospitals (7.5%) in Ethiopia [20]. This could be explained due to the difference in the quality of obstetric care across health facilities. Furthermore, the utilization of retrospective record review rather than primary data may increase the possibility of missing-out near-miss cases due to poor documentation. Compared with other studies done out of Ethiopia, this MNM is almost lower than the finding of a study conducted in India [21] but higher than many African and Asian countries [22–28] suggesting that Ethiopia is still having the highest burden of maternal morbidity and mortality.

This study found that severe preeclampsia was the leading near-miss event (31%) followed by severe postpartum hemorrhage (26%). This is similar to the national estimation of MNM in Ethiopia [9], findings of different studies performed elsewhere in Ethiopia [4, 19, 20] and outside Ethiopia [28]. Similarly, an unmatched case-control study from Nekemte Referral Hospital in 2018 also reported hypertensive disorder during pregnancy as the highest cause of MNM followed by obstetric hemorrhage [29]. Besides, studies from Kefa, Benchi Maji, and Sheka hospitals of South West Ethiopia [17] and Jimma University Hospital [16] reported uterine rupture (27%), followed by hypertensive disorders (24%) and obstetric hemorrhage (24%) as the leading near-miss events. As reported by this and the majority of other recent studies, hypertensive disorder during pregnancy becomes the leading cause of MNM than postpartum bleeding. This change in proportion is an expected event since hemorrhage could be reduced because of the provision of misoprostol and other preventive measures at a community level, increased community awareness, and clinicians' due attention to it. On the contrary, there could be an increase in hypertensive disorders during pregnancy due to an increase in the prevalence of hypertension, low preventive measures at the community level, and comparatively lower community awareness of these problems in Ethiopia [30, 31].

The multivariate logistic regression analysis of this study indicated that the site of the study (place and type of hospital where participants were admitted for maternal health services) was statistically associated with MNM. Participants who were admitted to Debre Berhan Referral Hospital and Enat General Hospital were five and four times more likely to develop MNM compared to participants who were admitted to Mehal Meda Primary Hospital. This difference could be due to the simple fact that the referral and general hospitals are more likely to admit women with severe pregnancy conditions as referred from the lower health facilities. The other possible reason is that these hospitals might not have a good quality of health service. Since nearly 70% of the near-miss cases have no referral history, it is also possible to speculate that mothers who attend their delivery at those general and referral hospitals are more likely to develop life-threatening pregnancy complications. A high incidence of MNM developing during hospitalization may be a good indicator of poor quality of care within facilities. High patient load, limited infrastructure, and shortage of trained skilled health personnel in the health facilities may compromise the quality of care.

Another clinically important variable that determines the occurrence of MNM was the utilization of partograph. Deliveries attended without using partograph are at a double odd of developing MNM compared with those attending with the use of partograph. Partograph helps health professionals to monitor wellbeing and progress in labor and indicate timely intervention when required. However, in this study, 38.3% of deliveries have no filled partograph forms in their delivery record. The lack of partograph use is an indicator of poor follow-up of labor, poor recording of maternal and labor conditions, delayed decision-making, and provision of interventions for the prevention of life-threatening pregnancy complications [32].

It is well documented that a history of previous pregnancy complications is a risk factor for the development of near-miss conditions. In this study, participants who had a history of pregnancy complications were seven times more likely to develop MNM as compared to participants who had no history of pregnancy complications. Abortion was one of the previous complications that increase the risk of developing MNM. Participants who had a history of abortion were 2.5 times more likely to develop MNM compared to participants who had no history of abortion. Abortion could be a potential risk factor for the development of near-miss conditions in subsequent pregnancies mainly by shortening the interpregnancy interval [33]. Previous studies showed that shortening of this interpregnancy interval may increase the risk of preeclampsia and uterine bleeding [33]. History of prior stillbirth risk factors that were independently associated with adverse perinatal outcomes [4] and previous cesarean section and having preexisting chronic medical disorder (Anemia or chronic hypertension) were significantly associated with MNM [34, 35].

Generally, this study provides insight into the current and six-year trends of MNM in three selected hospitals in Central Ethiopia. It also identifies the leading cause of MNM across time, which is helpful for prioritized interventions. While taking these sightings, it is good to consider some limitations of this study. First, there is a possibility of over- or underestimation or misdiagnosis of MNM due to the poor registration of patient records in the patient's card and delivery registration. For example, a physician may say “mild preeclampsia” (not a sign of near-miss), while there is also a record showing the mother taking magnesium sulfate (an indication of severe preeclampsia) which confuses the conclusion of diagnosis. Second, this poor recording also creates difficulties to confirm the presence or absence of organ dysfunction and life-saving interventions. This is primarily due to the absence of recorded diagnostic results that could suggest the presence or absence of organ dysfunction. Another limitation of this study is that it could not analyze all possible risk factors because it solely relied on secondary data (a patient's medical record) which missed socioeconomic variables of MNM.

## 5. Conclusions

Even though lower than the national figure, the proportion of MNM in Debre Berhan, Mehal Meda, and Enat hospitals was very high, and there was no change in the last six consecutive years. Complications of hypertensive disorder during pregnancy (severe preeclampsia and eclampsia) accounted for nearly half (47%) of the total life-threatening pregnancy complications. The place and type of hospital where the participants were admitted, use of partograph, history of abortion, and history of pregnancy complications were factors associated with MNM. Therefore, the zonal health department and hospital managers should work on the prevention and early detection of pregnancy complications before becoming life-threatening conditions. Hypertensive disorders of pregnancy and obstetric hemorrhage were the two main causes of near-misses that require strict and quick management protocols. To reduce the burden of severe maternal outcomes in the study area and Ethiopia, there is a need for timely management of life-threatening pregnancy complications and improved access to essential emergency obstetric care interventions. Prevention of hypertensive disorder of pregnancy, partograph use during labor follow-up, and close monitoring of women who had a history of abortion and any pregnancy complications are recommendations that need special emphasis. Researchers who want to estimate MNM are recommended to perform research aimed at registration and diagnostic procedures than routine medical records, while primary data from mothers are preferred for factor analysis.

## Figures and Tables

**Table 1:** Reproductive history of mothers admitted to selected hospitals in North Shewa, Central Ethiopia, from 2012 to 2017 (*n* = 905).

Variables	Frequency^∗^ (%)
Age of mothers	
<20 years old	84 (9.3)
20-34 years old	667 (73.7)
≥35 years old	154 (17.0)

Gravidity (*n* = 901)	
1	459 (50.9)
2-4	313 (34.7)
≥5	129 (14.3)

Parity (*n* = 901)	
0	437 (48.5)
1-2	296 (32.9)
3-4	94 (10.4)
≥5	74 (8.2)

Previous history of abortion (*n* = 893)	
Yes	54 (6.0)
No	839 (94.0)

Previous history of stillbirth (*n* = 898)	
Yes	40 (4.5)
No	858 (95.5)

Previous history of any pregnancy complications (*n* = 887)	
Yes	77 (8.7)
No	810 (91.3)

Previous history of cesarean section	
Yes	28 (3.1)
No	862 (96.9)

Type of current pregnancy (*n* = 897)	
Single	865 (96.4)
Twin	32 (3.6)

Antenatal care status (*n* = 875)	
No	276 (30.5)
Yes	599 (66.5)

Number of ANC visits (*n* = 494)	
ANC 1	85 (17.2)
ANC 2	110 (22.3)
ANC 3	129 (26.1)
4 and above	170 (34.4)

The final mode of delivery (*n* = 534)	
Vaginal delivery	446 (83.5)
Cesarean section	73 (13.7)
Laparotomy	15 (2.8)

Partograph utilization (*n* = 890)	
Not used	341 (38.3)
Used	549 (61.7)

Referral history (*n* = 886)	
No	684 (77.2)
Yes	202 (22.8)

^∗^Sum of frequencies did not fit the total sample size due to missing values.

**Table 2:** Factors associated with the occurrence of the maternal near-miss case among mothers delivered in selected Hospitals of North Shewa Zone, Central Ethiopia (2012-2017).

Variables	Maternal near-miss		COR (95% CI)	AOR (95% CI)
	Yes (%)	No (%)		
Hospital name				
Mehal Meda Primary Hospital	8 (4.85)	157 (95.15)	1.00	1.00
Enat General Hospital	34 (11.81)	254 (88.19)	2.63 (1.19, 5.82)	3.76 (1.35, 10.3)
Debre Berhan Referral Hospital	87 (19.25)	365 (80.75)	4.68 (2.21, 9.88)	4.85 (1.82, 12.94)

Age of mother				
15-19	8 (9.5)	76 (90.5)	1.00	1.00
20-34	94 (14.1)	573 (85.9)	1.6 (0.73-3.3)	1.4 (0.75-2.6)
≥35	27 (17.5)	127 (82.5)	2.0 (0.8-4.7)	2.2 (0.78-6.0)

Gravidity				
1	46 (10.02)	413 (89.98)	1.00	1.00
2-4	54 (17.25)	259 (82.75)	1.87 (1.24, 2.7)	1.7 (1.05, 2.9)
≥5	27 (20.93)	102 (79.07)	2.38 (1.4, 4.0)	2.0 (1.01, 4.1)

Parity				
0	48 (10.98)	389 (89.02)	1.00	1.00
1-2	42 (14.19)	254 (85.81)	1.34 (0.9, 2.1)	1.55 (0.6, 4.32)
3-4	24 (25.53)	70 (74.47)	2.78 (1.6, 4.8)	2.91 (0.8, 10.6)
≥5	13 (17.57)	61 (82.43)	1.73 (0.9, 3.4)	1.75 (0.35, 8.719)

Use of partograph				
Used	36 (9.73)	334 (90.27)	1.00	1 : 00
Not used	88 (16.92)	432 (83.08)	1.9 (1.3, 2.9)	1.89 (1.17, 3.04)

History of stillbirth				
Yes	10 (25.00)	30 (75.00)	2.11 (1.01, 4.4)	1.01 (0.38, 2.7)
No	117 (13.64)	741 (86.36)	1.00	1.00

History of abortion				
Yes	17 (31.48)	37 (68.52)	3.11 (1.7, 5.7)	2.52 (1.18, 5.37)
No	108 (12.87)	731 (87.13)	1.00	1 : 00

History of any pregnancy complications				
Yes	38 (49.35)	39 (50.65)	8.1 (4.9, 13.3)	6.91 (3.89, 12.28)
No	87 (10.74)	723 (89.26)	1.00	1 : 00

COR stands for crude odds ratio, while AOR is for adjusted odds ratio and CI is for a confidence interval.

## Data Availability

The SPSS dataset used to support the findings of this study can be released upon application to the principal investigator who can be contacted at tsfbrm@gmail.com. This was revised in the final version of the manuscript. But, at this time, we did not deposit the dataset.

## Abbreviations

ANC: Antenatal care
AOR: Adjusted odds ratio
MNM: Maternal near-miss
OR: Odds ratio
SAMM: Severe acute maternal morbidity
SSA: Sub-Saharan Africa
WHO: World Health Organization.

## Supplementary Materials

Supplementary Materials Supplementary Material 1: filled STROBE statement—checklist of items that should be included in reports of cross-sectional studies.
