# Supplementary material for: Trends of and Factors Associated with Maternal Near-Miss in Selected Hospitals in North Shewa Zone, Central Ethiopia
Source: J Pregnancy. 2022 Sep 9;2022:2023652. doi: 10.1155/2022/2023652 (PMC9481311; doi:10.1155/2022/2023652)
Supplement: Supplementary Materials — Supplementary Material 1: filled STROBE statement—checklist of items that should be included in reports of cross-sectional studies. [file 2023652.f1.doc]

**STROBE Statement—Checklist of items that should be included in reports of *cross-sectional studies* *[Trends of and factors associated with maternal near-miss in selected hospitals in North Shewa Zone, Central Ethiopia]***

|  | Item No | Recommendation | Line # |
| --- | --- | --- | --- |
| **Title and abstract** | 1 | (*a*) Indicate the study’s design with a commonly used term in the title or the abstract | Line 22 |
|  |  | (*b*) Provide in the abstract an informative and balanced summary of what was done and what was found | Lines 18 to 41 |
| Introduction | | |  |
| Background/rationale | 2 | Explain the scientific background and rationale for the investigation being reported | Lines 44-110 |
| Objectives | 3 | State-specific objectives, including any prespecified hypotheses | 104-107 |
| Methods | | |  |
| Study design | 4 | Present key elements of study design early in the paper | Method, line 113 |
| Setting | 5 | Describe the setting, locations, and relevant dates, including periods of recruitment, exposure, follow-up, and data collection | Method lines 113-126 |
| Participants | 6 | (*a*) Give the eligibility criteria and the sources and methods of selection of participants | Method lines 128-131 |
| Variables | 7 | Clearly define all outcomes, exposures, predictors, potential confounders, and effect modifiers. Give diagnostic criteria, if applicable | Method lines 146-158 |
| Data sources/ measurement | 8* | For each variable of interest, give sources of data and details of methods of assessment (measurement). Describe comparability of assessment methods if there is more than one group | Method lines 146-158 & 160-168 |
| Bias | 9 | Describe any efforts to address potential sources of bias | Method lines 160-179 |
| Study size | 10 | Explain how the study size was arrived at | Method lines 131-135 |
| Quantitative variables | 11 | Explain how quantitative variables were handled in the analyses. If applicable, describe which groupings were chosen and why | Method lines 171-179 |
| Statistical methods | 12 | (*a*) Describe all statistical methods, including those used to control for confounding | Method lines 173-178 |
|  |  | (*b*) Describe any methods used to examine subgroups and interactions | NA |
|  |  | (*c*) Explain how missing data were addressed | Method lines 169-171 |
|  |  | (*d*) If applicable, describe analytical methods taking into account of sampling strategy | Method lines 171-178 |
|  |  | (*e*) Describe any sensitivity analyses | NA |
| Results | | |  |
| Participants | 13* | (a) Report numbers of individuals at each stage of study—e.g., numbers potentially eligible, examined for eligibility, confirmed eligible, included in the study, completing follow-up, and analysed | The results line 181-273 |
|  |  | (b) Give reasons for non-participation at each stage | The results line 182-183 |
|  |  | (c) Consider the use of a flow diagram | NA |
| Descriptive data | 14* | (a) Give characteristics of study participants (eg demographic, clinical, social) and information on exposures and potential confounders | The result, table 1 lines 182-229 |
|  |  | (b) Indicate the number of participants with missing data for each variable of interest | 236-238, Table 1 |
| Outcome data | 15* | Report numbers of outcome events or summary measures | The result lines 239-270; 286-304, Table 2 |
| Main results | 16 | (*a*) Give unadjusted estimates and, if applicable, confounder-adjusted estimates and their precision (eg, 95% confidence interval). Make clear which confounders were adjusted for and why they were included | The result, line 195-229; Table 2, Figure 1-2 |
|  |  | (*b*) Report category boundaries when continuous variables were categorized | Result, Table 1 & 2 |
|  |  | (*c*) If relevant, consider translating estimates of relative risk into absolute risk for a meaningful period | NA |
| Other analyses | 17 | Report other analyses done—eg analyses of subgroups and interactions and sensitivity analyses | NA |
| Discussion | | |  |
| Key results | 18 | Summarise key results concerning study objectives | Discussion, Paragraphs 1, 2, & 6 lines 251-270 and 311-324 |
| Limitations | 19 | Discuss limitations of the study, taking into account sources of potential bias or imprecision. Discuss both the direction and magnitude of any potential bias | Strengths and limitations lines 346-358 |
| Interpretation | 20 | Give a cautious overall interpretation of results considering objectives, limitations, the multiplicity of analyses, results from similar studies, and other relevant evidence | Discussion, Paragraphs 3-5, lines 274-345 |
| Generalisability | 21 | Discuss the generalisability (external validity) of the study results | Strengths and limitations lines 346-358 |
| Other information | | |  |
| Funding | 22 | Give the source of funding and the role of the funders for the present study and, if applicable, for the original study on which the present article is based | NA |

*Give information separately for exposed and unexposed groups.

**Note:** An Explanation and Elaboration article discusses each checklist item and gives methodological background and published examples of transparent reporting. The STROBE checklist is best used in conjunction with this article (freely available on the Web sites of PLoS Medicine at http://www.plosmedicine.org/, Annals of Internal Medicine at http://www.annals.org/, and Epidemiology at http://www.epidem.com/). Information on the STROBE Initiative is available at www.strobe-statement.org.
